# An In-Depth Review of Molecularly Imprinted Electrochemical Sensors as an Innovative Analytical Tool in Water Quality Monitoring: Architecture, Principles, Fabrication, and Applications

**DOI:** 10.3390/mi16030251

**Published:** 2025-02-23

**Authors:** Mbuyamba Divin Mukendi, Oluseyi Sikiru Salami, Nomvano Mketo

**Affiliations:** Department of Chemistry, College of Science, Engineering and Technology (CSET), University of South Africa, The Science Campus, Florida Park, Corner Christian de Wet and Pioneer Avenue, Florida 1709, South Africa; 11130342@mylife.unisa.ac.za (M.D.M.); 62325019@mylife.unisa.ac.za (O.S.S.)

**Keywords:** MIPs, electrochemical sensors, analytical tool, WQM, architecture, principles, fabrication, applications

## Abstract

Molecularly imprinted electrochemical sensors (MI-ECSs) are a significant advancement in analytical techniques, especially for water quality monitoring (WQM). These sensors utilize molecular imprinting to create polymer matrices that exhibit high specificity and affinity for target analytes. MI-ECSs integrate molecularly imprinted polymers (MIPs) with electrochemical transducers (ECTs), enabling the selective recognition and quantification of contaminants. Their design features template-shaped cavities in the polymer that mimic the functional groups, shapes, and sizes of target analytes, resulting in enhanced binding interactions and improved sensor performance in complex water environments. The fabrication of MI-ECSs involves selecting suitable monomeric units (monomers) and crosslinkers, using a target analyte as a template, polymerizing, and then removing the template to expose the imprinted sites. Advanced methodologies, such as electropolymerization and surface imprinting, are used to enhance their sensitivity and reproducibility. MI-ECSs offer considerable benefits, including high selectivity, low detection limits, rapid response times, and the potential for miniaturization and portability. They effectively assess and detect contaminants, like (toxic) heavy metals (HMs), pesticides, pharmaceuticals, and pathogens, in water systems. Their ability for real-time monitoring makes them essential for ensuring water safety and adhering to regulations. This paper reviews the architecture, principles, and fabrication processes of MI-ECSs as innovative strategies in WQM and their application in detecting emerging contaminants and toxicants (ECs and Ts) across various matrices. These ECs and Ts include organic, inorganic, and biological contaminants, which are mainly anthropogenic in origin and have the potential to pollute water systems. Regarding this, ongoing advancements in MI-ECS technology are expected to further enhance the analytical capabilities and performances of MI-ECSs to broaden their applications in real-time WQM and environmental monitoring.

## 1. Introduction

Water is the most vital natural resource for all human activities. Still, its quality is deteriorating due to contamination from expanding contaminants, brought on by population growth, industry, agriculture, and urbanization, among other factors [1,2,3]. The requirements to monitor and maintain high water quality (WQ) are crucial and urgent tasks for the global need, as mandated in the United Nations World Water Development Reports (UNWWDRs) [4,5,6], following the World Health Organization (WHO) guidelines on water sustainability and criteria for WQ [7].

Maintaining ecosystem health and the population’s livelihood requires identifying the water contaminants that are causing pollution using WQ detection strategies. These strategies sometimes involve a tedious, time-consuming, expensive procedure, manual laboratory analyses, statistical assessments, and advanced analytical tools. However, the use of modern analytical technologies or tools, such as electrochemical sensors (ECSs) [8,9,10], may assist and could lessen the concerns about water quality monitoring (WQM), as seen in the case of the adoption of molecularly imprinted electrochemical sensors (MI-ECSs) for proper WQM and treatment [3,11,12,13,14].

**Figure 1:**
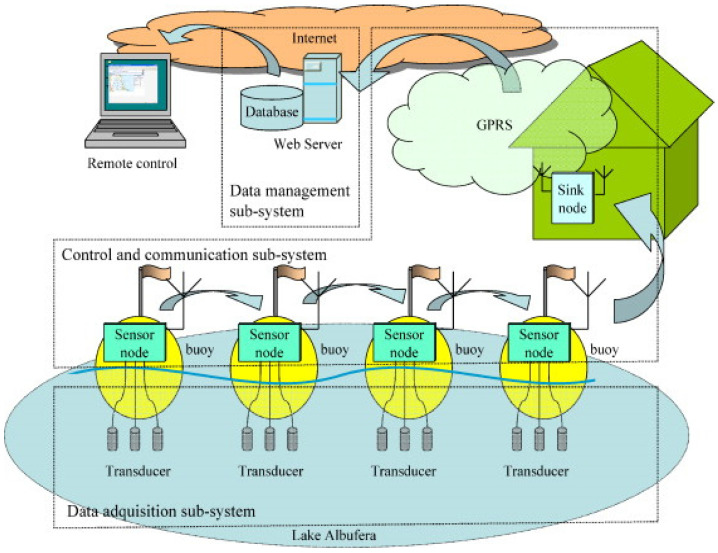
Real-time system for WQM. Reprinted from [18], Copyright (2010), with permission from Elsevier.

Regarding these concerns about the development of efficient water monitoring and treatment strategies to achieve high quality and availability of water for safe use [1,15], some online, real-time WQM devices have been developed to analyze some WQ parameters, such as chloride (Cl^−^), ammonium (NH_4_^+^), and nitrate (NO_3_^−^) concentration levels, as well as pH, temperature, and dissolved oxygen levels, among others, in water systems [3,16,17,18]. As illustrated in Figure 1, a wireless sensor network method with an online water analytical system consisting of fourteen (14) buoys was proposed in [18]. Each buoy, equipped with sensors, mainly ion-selective electrodes (ISEs), to measure Cl^−^, NH_4_^+^, and NO_3_^−^ concentrations, was installed in a freshwater lake. These buoys communicated wirelessly using secondary data transmission services, such as GPRS (General Packet Radio Services) or GSM (Global System for Mobile Communications), allowing the data from all the buoys to be aggregated in one location. The collected data was accessible online, enabling real-time water system monitoring and control.

The aforementioned online water analysis reveals the demand for an ever-expanding array of WQM techniques, spanning from traditional methods to emerging technologies, such as the MI-ECS system, which demonstrate the high selectivity and sensitivity of their sensors for the detection of various contaminants in water systems [1,9,12,13,14,19].

### 1.1. Historical Development and Evolution of MI-ECSs

The concept of today’s molecularly imprinted technology (MIT) was introduced in the 1970s by Wulff and Sarhan [20], who demonstrated the feasibility of preparing synthetic polymers with predetermined selectivity for target molecules. Early applications were limited due to challenges in achieving high binding affinities and specificities. However, advancements in polymer chemistry and fabrication techniques in the 1990s led to significant improvements. Integrating molecularly imprinted polymers (MIPs) with ECSs marked a pivotal development, enabling MI-ECSs to be created with enhanced analytical performance [21].

The evolution of MI-ECSs has been driven by innovations in imprinting techniques, such as surface imprinting and electropolymerization, which have addressed issues related to template removal and binding site accessibility [9,13]. Furthermore, advances in nanomaterials [9,21,22,23], such as nanoparticles and nanocomposites [9], have improved the sensors’ sensitivity and selectivity. Today, MI-ECSs are employed in various fields [24], including environmental monitoring [9,12], such as WQM [3,9], clinical diagnostics [8,25], and food safety [25], among others, showcasing their versatility and potential for future applications [11,20,21,22,23,24,25,26,27,28,29].

**Figure 2:**
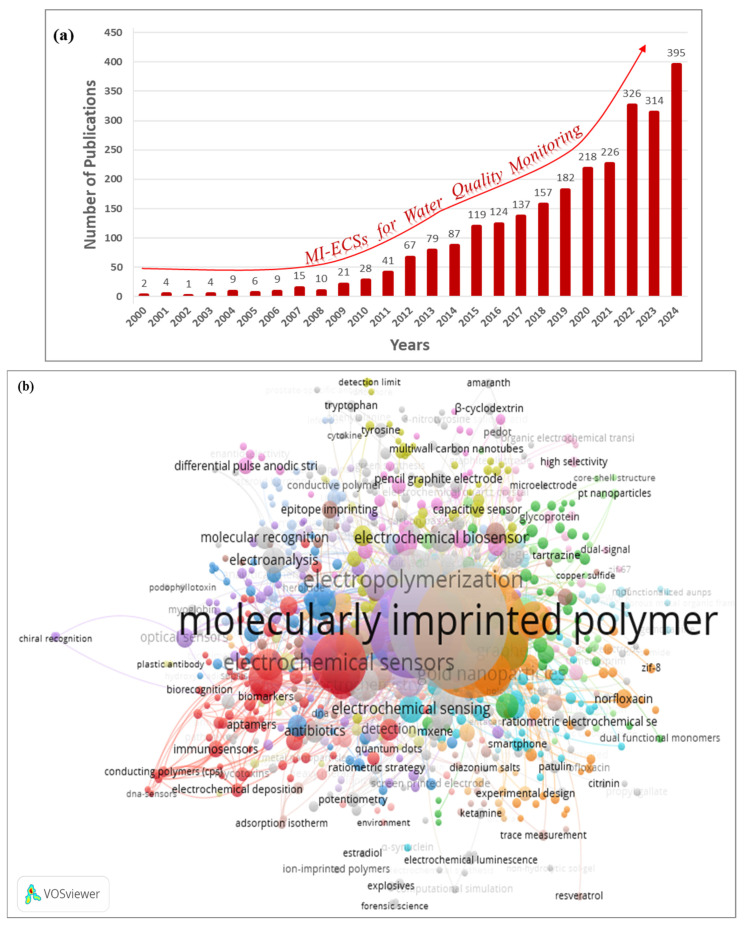
(**a**) Several publications made on using electrochemical sensors (including MI-ECSs) for real-time WQM and (**b**) the bibliometric analysis of various applications of MI-ECSs in WQM, performed using VOSviewer software version 1.6.20 to analyze the past two decades. Search keywords: “MI-ECSs and water quality monitoring”. Scopus (December 2024).

The utilization of MI-ECSs in WQM has significantly expanded. Early research laid the foundation for these sensors’ development, emphasizing their high specificity and sensitivity in detecting contaminants [19]. Over the decades, advancements in materials science, nanotechnology, and particularly electrochemistry have fueled their adoption, leading to a marked increase in publications, as illustrated in Figure 2a. A bibliometric analysis, presented in this review (Figure 2b), sheds further light on this, using VOSviewer software version 1.6.20 and the Scopus database, focusing on the keywords MI-ECSs and WQM. This surge highlights the growing recognition of MI-ECSs as a critical analytical tool for detecting contaminants, ensuring water safety, and addressing environmental challenges [24,25]. 

### 1.2. Importance of MI-ECSs

**Figure 3:**
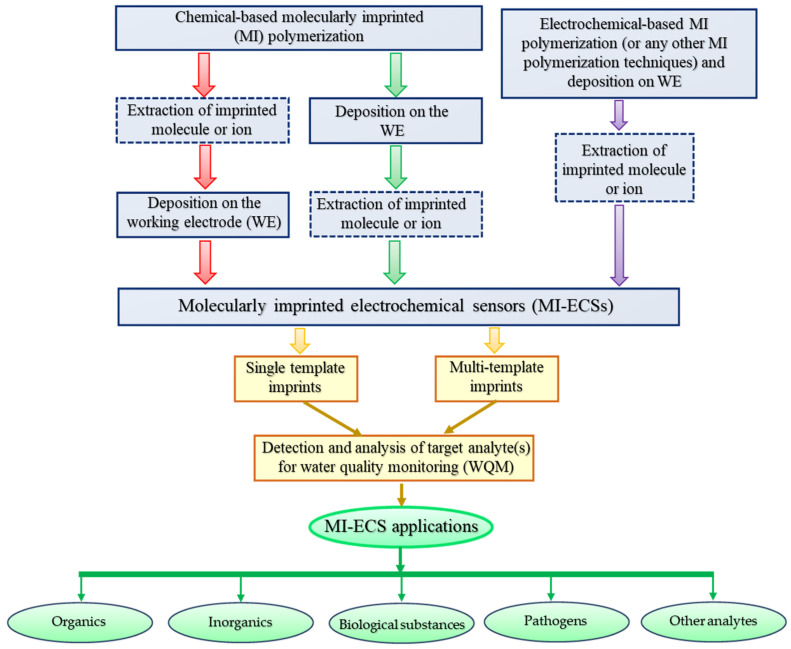
A flowchart showing the summary of MI-ECS development and its possible applications in WQM.

Molecularly imprinted electrochemical sensors (MI-ECSs) have become indispensable in contemporary analytical chemistry, based on their high selectivity, specificity, versatility, and sensitivity in detecting a wide range of analytes. These sensors excel in complex matrices, providing accurate, real-time, and on-site analyses without the need for extensive sample preparation, a significant advantage over conventional analytical techniques, like spectroscopy, mass spectrometry, chromatography, and immunoassays [13,19,23]. MI-ECSs offer faster response times and lower detection limits, making them suitable for environmental monitoring, clinical diagnostics, and food safety applications [30]. Their cost-effectiveness and potential for miniaturization and portability further enhance their applicability and utility in various fields, positioning them as a transformative analytical tool [3,13,21]. MI-ECSs are applicable for detecting a range of water quality parameters (WQPs), including organic and inorganic pollutants, pathogens and their associated toxins, and various other biological substances, such as hormones and enzymes, among others. Figure 3 depicts a flowchart as a summary for a better understanding of MI-ECS development and its possible applications in WQM.

### 1.3. Water Quality Parameters Signaling Presence of Contaminants and Requirements for WQM

**Figure 4:**
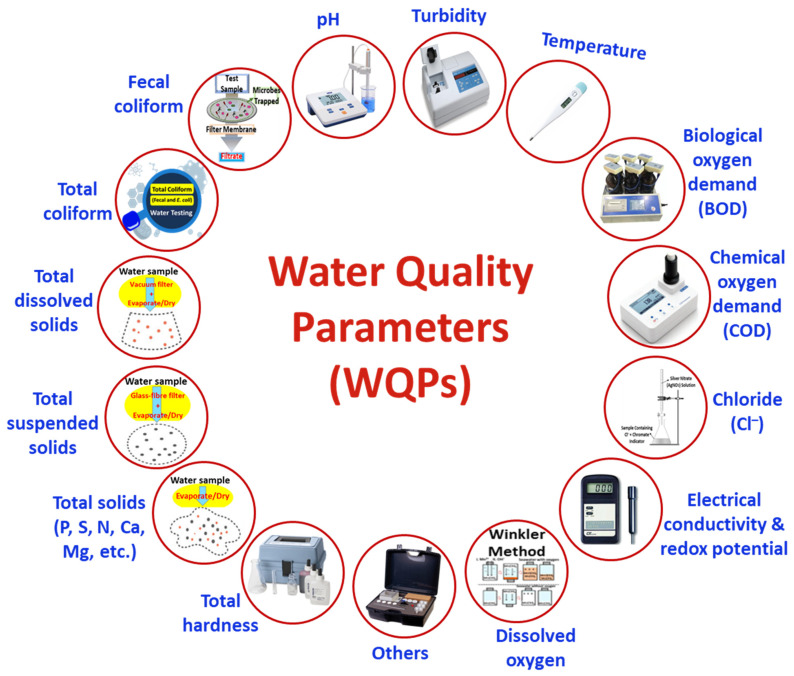
WQ parametric requirements for WQM.

The monitoring of water quality by MI-ECSs is facilitated by noting the level and status of some other key WQPs, which serve as a key measurable signal for the presence of contaminants in water bodies, the requirements for WQM, and as a guide for water treatment [1,31]. These key WQPs include the pH, temperature, turbidity, biological oxygen demand (BOD), total suspended solids (TSSs), particulate phosphorus, nitrates, fecal coliform, and chemical oxygen demand (COD), among others [1,32], as illustrated in Figure 4. These parameters are concerned with the water monitoring [1] and treatment strategies [15,31] that are used to achieve the high quality and availability of water for safe use. For example, high turbidity suggests the presence of contaminants, like inorganic and organic substances, in water, and it is correlated to the total hardness, electrical conductivity, sulfates, total dissolved solids (TDSs), COD, and total coliforms (including fecal coliforms), which indicate potential pathogenic microorganisms, like bacteria, in the water [1].

Regarding this, an ever-expanding array of WQM techniques is demanded, spanning from traditional methods to emerging technologies, such as the MI-ECS system [1,9,12,13,14,19].

### 1.4. Objectives of the Review

The specific objectives of this review on MI-ECSs as an innovative analytical tool in WQM are four-fold. These include


**Review’s Major Objectives**

**Section**

–To elucidate the architectural design of MI-ECSs, highlighting the integration of MIPs with electrochemical transducers (ECTs) and their implications for sensor performance.
2
–To expound on the underlying principles governing MI-ECS functionality, based on target analyte selective recognition and transduction mechanisms.
3
–To detail the fabrication processes of MI-ECSs, including the selection of monomers, template molecules, and advanced imprinting techniques in detecting contaminants in water.
3
–To explore various the reported applications of MI-ECSs in WQM.
4

## 2. Architecture of MI-ECSs

### 2.1. Electrochemical Cell Key Components and Modifications

**Figure 5:**
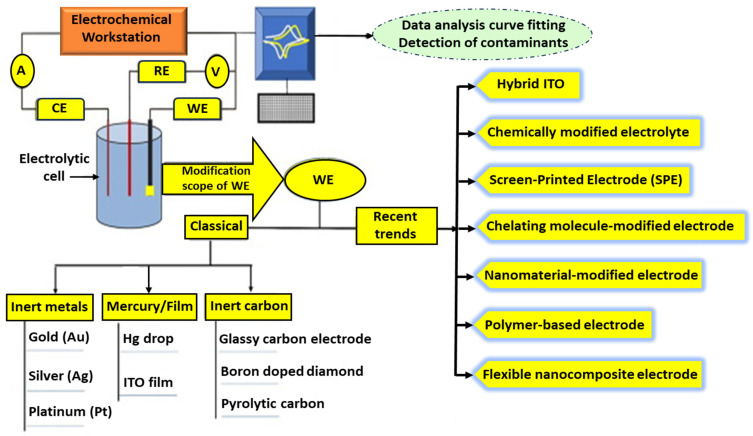
Electrochemical cell system with important components: A—ammeter; V—voltmeter; and ITO—indium tin oxide. Reprinted from [34] with some modifications, Copyright (2021), with permission from Elsevier.

Besides MIPs, the architecture of MI-ECSs is fundamentally centered around the three key electrode components of electrochemical (EC) cells: the reference electrode (RE), working electrode (WE), and auxiliary or counter electrode (AE/CE) [26], as illustrated in Figure 5. The WE is the site of the primary electrochemical reaction (the so-called redox reaction between the electrode material and the electrolyte). Due to the great influence of the WE on the electrochemical sensitivity of the MI-ECS system, it is usually modified with MIPs to provide selective binding sites for target analytes.

Common materials for the WE include carbon-based electrodes, such as glassy carbon electrodes (GCEs), graphene, and carbon paste, due to their high conductivity, stability, and ease of modification [33]. Metal electrodes, such as gold (Au) and platinum (Pt), are also popular for their excellent electron transfer characteristics and biocompatibility, especially in biosensing applications. The RE, which keeps a stable potential, usually comprises materials like Ag/AgCl or saturated calomel, which offer stable and reproducible reference potentials. The CE, typically made from inert materials like Pt or carbon (C) (graphite), completes the electrochemical circuit (ECC) by allowing current to flow through the cell without being involved in the reaction [34].

The choice of materials for the electrodes, membranes, and coatings further enhances the performance of MI-ECSs. The electrode surface is often modified with MIPs [12,13,28,34], nanomaterials (e.g., Au nanoparticles (AuNPs), carbon nanotubes (CNTs)) [8,9,23,34], or conducting polymers to increase their sensitivity and selectivity [29,30,34]. Membranes such as Nafion or cellulose acetate are employed to prevent fouling [35], improve ion selectivity [34], and enhance sensor stability [36]. Coatings, including polyaniline (PANI) [33] or polypyrrole (PPy) [37,38], are employed to enhance the mechanical strength, conductivity, and biocompatibility of MI-ECSs, and these are crucial for applications in complex biological environments [12,13,14,25,33,39].

### 2.2. Electrochemical Sensing Techniques and Configurations

The electrochemical sensors (ECSs), mainly amperometric, voltammetric, and electrochemical impedance spectroscopic (EIS) sensors, utilized in MI-ECS systems are characterized by their simplicity, high sensitivity, accuracy, and robustness [14,40,41]. Other sensing techniques include potentiometry and conductometry [41]. Each of these sensors is tailored to the specific detection requirements of WQM [14]. Recent innovations have allowed for multiple in situ measurements, enhancing their use in diverse environmental, biological, and chemical applications, especially for identifying harmful substances in water for WQ management [3,14].

**Figure 6:**
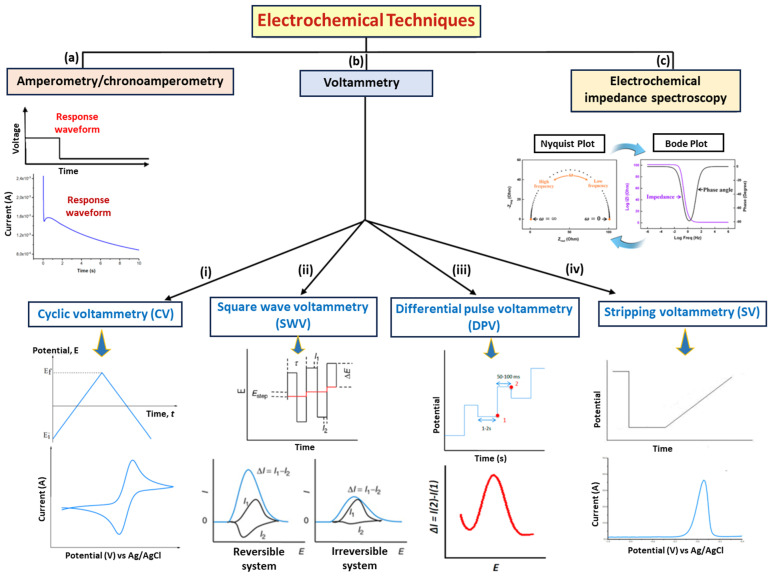
Schematic illustration of commonly used electrochemical sensing techniques and configurations in WQM: (**a**) amperometric/chronoamperometric configuration, (**b**) voltammetric configuration and its various types ((i) CV, (ii) SWV, (iii) DPV, and (iv) SV), and (**c**) EIS configuration [14].

Based on the molecular imprinting strategies adopted, these sensors can be customized and modified to detect various ions, molecules, inorganics, and organics, irrespective of electroactivity. For targets that are not electroactive, MI-ECS methods depend on modulating the electrochemical signals of a probe immersed in the solution. This is illustrated in Figure 5 and Figure 6.

#### 2.2.1. Amperometric Sensors

By applying a specific potential across the electrode system, amperometric sensors measure the generated current as a function of time due to redox reactions at the WE surface on binding a target analyte to the MIP coated on the WE. As illustrated in Figure 6a, this current is linearly dependent on the concentration of the analyte, and it is plotted against time. This makes amperometric or chronoamperometric MI-ECSs ideal for detecting trace contaminants, like heavy metals and organic pollutants, in sample matrices, such as water systems [14,40].

Amperometric sensors exhibit rapid response times, excellent reproducibility, and high sensitivity, making them ideal for various applications. Their potential for miniaturization enables online WQ monitoring [42], leading to the development of most existing portable sensors like MI-ECSs.

#### 2.2.2. Voltammetric Sensors

Voltammetric sensors are diverse electrochemical sensing techniques used in water quality monitoring, each offering distinct advantages based on the target analyte and environmental conditions [14,40,41,43]. These sensing techniques (Figure 6b) include cyclic voltammetry (CV), square wave voltammetry (SWV), differential pulse voltammetry (DPV), linear sweep voltammetry (LSV), and stripping voltammetry (SV), as described below.

##### Cyclic Voltammetry (CV)

Cyclic voltammetry (CV) is a commonly employed voltammetric technique for quantitative analysis and studying the redox processes of electroactive species. It entails the cyclical adjustment of the WE’s potential, as illustrated in Figure 6b(i), aiding the analysis of the electrochemical behavior of various electroactive species in water and other matrices [43]. In the CV experiment, the total current measured is the sum of capacitive and faradaic currents, with the scan rate influencing the speed of the applied potential. Higher scan speeds decrease the size of the diffusion layer, resulting in a greater current. However, a large capacitive current might interfere with the more sensitive faradaic current, which is linearly related to the analyte concentration, reducing the technique’s sensitivity [40,41]. CV is typically employed to assess sensor surface conditions before and after functionalization. Potential pulse techniques, like DPV and SWV, are preferred for more accurate measurements, as they minimize capacitive current interference and enhance sensor sensitivity [14,40,41].

##### Square Wave Voltammetry (SWV)

Square wave voltammetry (SWV) is a pulse technique that provides more rapid analyses and enhanced sensitivity compared to DPV, making it ideal for the simultaneous detection of various contaminants in sample matrices, such as water systems. Figure 6b(ii) illustrates the application of the SWV technique pulses to both reversible and irreversible systems. The faradaic current (∆i) is determined by the difference between the currents measured after each potential step [40]. SWV is frequently combined with advanced nanomaterials to improve selectivity and lower detection limits, delivering accurate, real-time data vital for water quality management. However, issues like electrode fouling and matrix effects require ongoing optimization and calibration for reliable performance [40].

##### Differential Pulse Voltammetry (DPV)

Differential pulse voltammetry (DPV) improves sensitivity by superimposing a series of voltage pulses onto a linear potential sweep, making it highly effective for the detection of heavy metal (HM) ions at trace levels in water samples [14,40]. As illustrated in Figure 6b(iii), DPV involves applying an initial potential at which no faradaic reactions take place, followed by a linear slope potential of constant amplitude pulses. The measured current is derived from the difference between the currents recorded just before and after the pulse, resulting in a distinct peak for the electrochemical reaction. DPV offers greater sensitivity than linear sweep methods, as it minimizes capacitive current interference [14].

##### Linear Sweep Voltammetry (LSV)

Linear sweep voltammetry (LSV) is an EC sensing technique for investigating redox reactions and assessing the EC properties of materials by linearly varying the potential of a WE while measuring the resulting current [40]. This technique offers valuable information about the redox behavior of analytes, highlighting the potential for reduction and oxidation, and facilitating the assessment of concentrations, reaction kinetics, and various other electrochemical parameters [40]. The resulting current change, plotted as a function of the applied potential, produces a voltammogram that can be analyzed to extract valuable information about the electrochemical system under study.

##### Stripping Voltammetry (SV)

Stripping voltammetry (SV) is a highly sensitive electrochemical sensing technique used for WQM. It involves preconcentrating trace metals onto the WE surface, followed by a stripping step in which the accumulated metals are oxidized or reduced, producing a current peak, as illustrated in Figure 6b(iv). The height of the current peak is directly related to the concentration of the metal. This technique allows for the detection of very low concentrations of contaminants, like HMs, such as cadmium (Cd), lead (Pb), and mercury (Hg), in water, making it ideal for environmental monitoring and ensuring water safety [14,40]. The primary SV techniques utilized include (i) anodic SV (ASV), which is employed for the trace detection of metals, such as Pb, zinc (Zn), cadmium (Cd), and copper (Cu); (ii) cathodic SV (CSV), which is explored for the detection of ionic species, like sulfide (S^2−^), thiocyanate (SCN^−^), and cerium(III) (Ce^3+^); and (iii) adsorption SV (AdSV), which is often used for detecting trace levels of nickel (Ni), cobalt (Co), and certain organic compounds [14,38].

In general, voltammetric procedures expose the detection system to the electroactivity of analytes. Regarding this, real-time current monitoring is performed for electroactive molecules, generating optimal signals from faradaic currents during the oxidation or reduction of analytes bound to the MIP on the WE surface of MI-ECSs. This allows for a correlation between the analyte concentration and the measured current under controlled conditions. Moreover, by utilizing redox probes, such as [Fe(CN)_6_]^3−/4−^, it is possible to generate signals even for analytes that are not electroactive [41].

#### 2.2.3. Electrochemical Impedance Spectroscopic (EIS) Sensors

An electrochemical impedance spectroscopic (EIS) sensor assesses the resistance or impedance to an alternating current applied to the sensor. This impedance changes when an analyte binds to the MIP of an MI-ECS, providing detailed information about the analyte concentration and the interaction kinetics [41,44]. As illustrated in Figure 6c, the impedance spectrum with the function *Z*(*ω*), where *ω* (=2π*f*) is the radial frequency (measured in radians per second (rad s^−1^)) and *f* is the applied frequency (measured in hertz (Hz)), obtained from the EIS technique, can be displayed in two formats: (i) the “Nyquist plot”, which presents the real and imaginary components of *Z*(*ω*) using Cartesian coordinates, and (ii) the “Bode plot”, which shows both the phase and the logarithm of the total impedance (log *Z*(*ω*)) as functions of the logarithm of the frequency [41,44,45]. EIS sensors are highly sensitive and suitable for detecting low concentrations of various water contaminants, including pathogens and organic molecules, as well as serving as biomarkers for diseases [41,44].

#### 2.2.4. Potentiometric Sensors

In the potentiometric-based MI-ECSs, the sensors operate by measuring the difference in the potential between a WE coated with MIP and an RE, without passing any current, that is, in conditions of nearly zero current flow. The target analyte binding to the MIP alters the electrode potential, correlating with the analyte concentration. This configuration is beneficial for monitoring ions and other charged species in water samples [30,46]. Selectivity in MI-ECS devices is accomplished through membranes made of MIPs that include specific ion exchangers or neutral carriers [9,41]. These devices often utilize chemically sensitive field-effect transistors, like silicon chips, which respond to electric fields at their gate electrodes [9]. In the same vein, the analyte binding to the MIP alters the surface potential of the silicon chip, affecting the current and allowing for the monitoring of reaction rates [9,30].

In addition to sensors based on MIPs, potentiometric sensors, such as fluoride-ion-selective electrode (F-ISE) potentiometric sensors, have proven effective for measuring inorganic ions, including fluorides, in aqueous matrices, like aqueous solutions of toothpastes [47]. These sensors exhibit high sensitivity, efficiency, and rapid response times when enhanced with MIPs [48], making them valuable tools for WQM. Their ability to accurately quantify specific ions in aqueous environments also underscores their potential for environmental applications [46].

#### 2.2.5. Conductometric Sensors

Conductometry, as an electrochemical sensing technique, measures electrical conductivity based on the movement of positively and negatively charged ions toward the cathode and anode, respectively, resulting in the generation of current flow when an electric field is applied between the two electrodes immersed in an electrolyte solution [41]. To create an MIP-based conductometric sensor, an MIP must first be transformed into a membrane. However, conductivity is cumulative, and this limits its ability to distinguish between different ions and renders it the least sensitive of the electrochemical sensing methods. Subtle variations in ionic limiting equivalent conductance pose challenges in differentiating between species, and a high concentration of a particular ion can hinder the detection of other ions [41].

#### 2.2.6. Comparison of Electrochemical Sensing Techniques as Components of MI-ECS Systems

Drawing from the principles, configurations, and applications of electrochemical (EC) sensors utilized in MI-ECS systems, as detailed in Section 2.2, each technique possesses distinct advantages and limitations based on their selectivity, sensitivity, and response time. These dependent factors play a crucial role in choosing the appropriate sensor technique for specific monitoring requirements. By integrating the various EC sensing techniques, MI-ECS systems can significantly improve the evaluation of water quality by generating a wide range of electrochemical profiles that accurately represent real-time environmental conditions. A summary of the different types of EC sensing techniques and their key features is provided in Table 1.

### 2.3. Innovations in MI-ECS Architecture

MI-ECSs have emerged as essential tools for the sensitive and accurate detection of specific target analytes across various fields, including environmental monitoring [12], WQM [9], food safety [49], and biomedical applications [49]. Recent developments in their design—particularly using nanostructured materials [50], the integration of microfluidic technologies [51], and enhancements in wireless and portable sensor systems [18]—have significantly improved their performance, versatility, and practicality.

#### 2.3.1. Nano-Structured Materials

Utilizing nanostructured materials in MI-ECSs has significantly contributed to improvements in sensitivity and specificity. Nanomaterials such as CNTs, metal nanoparticles (MNPs), graphene (GR), and graphene oxide (GO), among others, provide high surface area-to-volume (*A*/*V*) ratios, which facilitate enhanced interactions between the target analyte and the surface of the sensor [52,53].

GR-based MI-ECSs are characterized by exceptional mechanical strength and electrical conductivity, facilitating faster electron transfer rates and reducing response times [54]. Moreover, the GR surface can be easily functionalized, allowing for the deposition of molecular imprints tailored for specific analytes, thereby enhancing selectivity [55]. For example, in a study carried out on a GR-functionalized MI-ECS, an MI-EC amperometric sensor was developed by Mao et al. [53] to detect dopamine (DA), in which a composite of graphite oxide sheets and Congo red-based molecularly imprinted polymers (GSCR-MIPs) was synthesized through free radical polymerization. This process involved methacrylic acid (MAA) as the functional monomer, ethylene glycol dimethacrylate (EGDMA) as the crosslinker, and 2,2’-azodiisobutyronitrile (AIBN) as the initiator. Under optimized experimental conditions, the sensor exhibited selective detection capabilities, an extensive linear concentration range from 10–830 μM, a detection limit of 0.1 μM, and excellent measurement repeatability. This type of MI-ECS represents a novel advancement for detecting and monitoring DA and other catecholamine drugs with similar molecular structures in aquatic environments.

**Figure 7:**
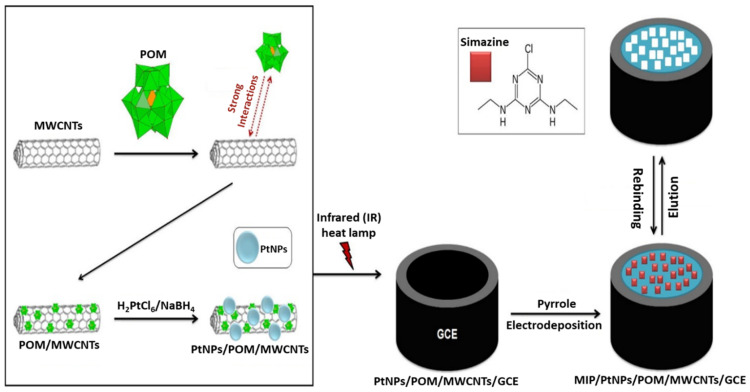
A schematic representation of the fabrication of an MI-VS based on MIP/PtNPs/POM/MWCNTs/GCE for detecting simazine in a water system. Reprinted from [57], Copyright (2016), with permission from Elsevier.

CNTs have also been employed to augment the performance of MI-ECSs [56]. The unique one-dimensional structure of CNTs not only promotes effective charge transport but also serves as a nanocarrier for molecular recognition sites. The integration of CNTs into MI-ECSs has demonstrated improved analytical performance due to their exceptional electronic properties and high stability [9]. For example, Ertan et al. [57] developed a molecularly imprinted voltammetric sensor (MI-VS) by applying a pre-prepared solution of polyoxometalate (POM)-functionalized multi-walled carbon nanotubes (MWCNTs) and platinum nanoparticles (PtNPs) onto a clean GCE for simazine detection in wastewater samples, as illustrated in Figure 7. The PtNPs enhanced the sensor’s effective active area and exhibited catalytic activity, significantly improving the performance of the MI-ECS compared to the MWCNT-only modified electrode. The method demonstrated a linear concentration range of 0.1 to 5.0 nM and a limit of detection (LOD) of 20 pM. Based on this, the sensor functions as an innovative MI-ECS for analyzing and monitoring water quality for a specific contaminant due to its high stability and reproducibility.

**Figure 8:**
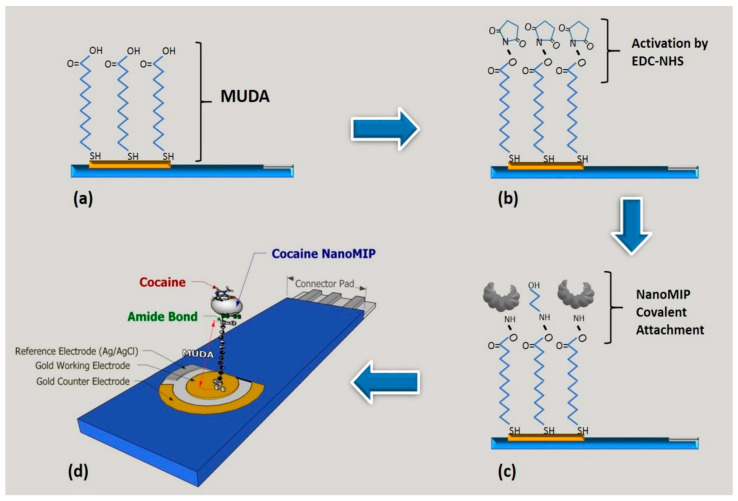
A schematic illustration of the covalent attachment of nanoMIPs on a gold (Au) screen-printed working electrode (WE) surface: (**a**) the formation of a self-assembly monolayer (SAM); (**b**) the activation of the carboxylic (COOH) group by EDC-NHS (1-ethyl-3-(3-dimethylaminopropyl) carbodiimide (EDC) and *N*-hydroxysuccinimide (NHS)); (**c**) the covalent attachment of nanoMIPs via amine coupling; and (**d**) the 3D model structure of the cocaine-detecting nanoMIPs ECS [59]. (MUDA: 11-mercaptodecanoic acid).

In addition, metallic nanoparticles, such as AuNPs, can enhance the electrochemical signal of MI-ECSs through localized surface plasmon resonance (SPR) effects, leading to improved LODs and sensitivity [58]. For instance, D’Aurelio et al. [59] developed a novel AuNP-functionalized MI-ECS for detecting trace cocaine levels in environmental samples, as illustrated in Figure 8. Their method used MIP-based nanoparticles (nanoMIPs) attached to gold electrodes, coupled with EIS. The sensor detected cocaine in a linear dynamic range of 100 pg/mL–50 ng/mL, with an LOD of 0.24 ng/mL, offering a sensitive, portable, and cost-effective approach for WQM and forensic applications.

#### 2.3.2. Integration with Microfluidics

The integration of MI-ECSs with microfluidic systems marks another significant innovation that enhances functionality and performance [60]. Microfluidics enable precise control over fluid dynamics at the microscale, allowing for the miniaturization of analytical processes and the concurrent detection of multiple analytes [61]. The microfluidic design facilitates efficient mass transport to the sensor surface, significantly reducing the consumption of reagents and enabling automated sample processing [60].

**Figure 9:**
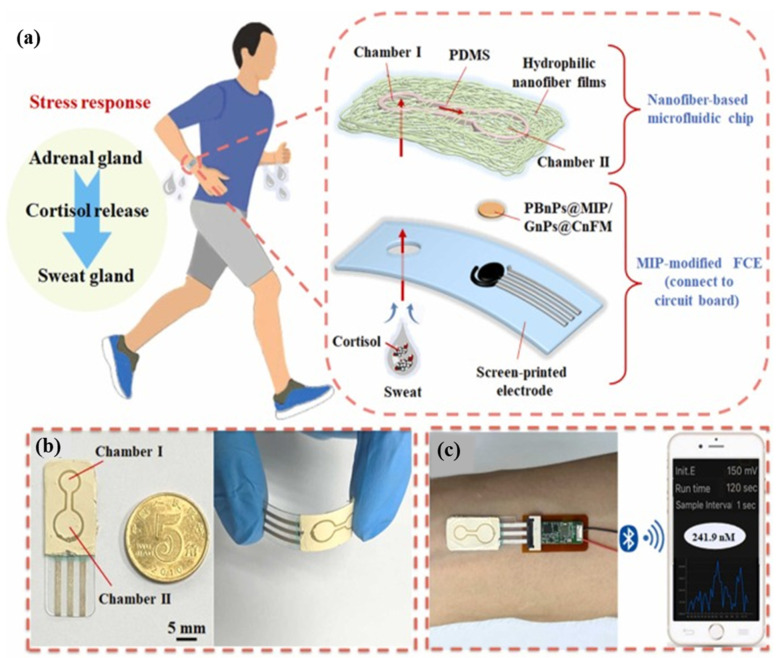
(**a**) A schematic illustration of a flexible, layered, MIP-based microfluidic ECS (a chip sensor) for detecting and analyzing the cortisol released in sweat and any other sources as a potential contaminant of water systems. (**b**) Photographs illustrating the sensor in both flat and bent positions. (**c**) Image showing the sensor integrated with a flexible circuit board on a volunteer’s wrist. (PDMS: polydimethylsiloxane; FCE: flexible carbon electrode). Reprinted from [60], with some modifications, Copyright (2023), with permission from Elsevier.

For instance, Mei et al. [60] developed a novel, flexible MI-ECS for in situ and real-time sweat detection and analysis, integrating microfluidics, nanofiber technology, and MIPs, as illustrated in Figure 9. Fabricated on a polyethylene terephthalate (PET) substrate, the sensor features an MIP-modified electrode for analyte detection and a nanofiber-based microfluidic layer for sweat transport. A gold nanoparticle-decorated carbon nanofiber membrane (GnPs@CnFM) enhances stability and sensitivity as a 3D matrix for the MIP, while embedded Prussian blue nanoparticles (PBnPs) act as a built-in redox probe for direct sweat monitoring. The sensor shows a broad detection range of 1 nM–1 μM with high selectivity and stability, and it serves as an innovative MI-ECS for monitoring cortisol in water systems or any aqueous media. 

Regarding this, integrating microfluidics with MI-ECSs facilitates real-time monitoring and feedback mechanisms, which can be critical in applications such as WQM in environmental settings [62]. This level of integration not only enhances the practicality of MI-ECSs but also opens avenues for further advancements in real-time technology for WQM.

#### 2.3.3. Wireless and Portable Sensor Systems

Innovations in wireless communication technologies have also transformed the architecture of MI-ECSs, making them more accessible and easier to deploy in the field [63]. The integration of wireless modules, such as Bluetooth and Wi-Fi, enables the real-time transmission of data from MI-ECSs to mobile devices or cloud-based platforms, facilitating remote monitoring and data analysis [18], as illustrated in Figure 1. This allows for remote monitoring and data transmission, facilitating immediate responses to pollution events without the need for specialized laboratory equipment [63].

The development of portable MI-ECSs has been particularly noteworthy. These systems are designed to be compact, lightweight, and powered by small rechargeable batteries or solar cells, rendering them suitable for remote or resource-limited environments [14,17]. Such capabilities are essential for applications in MQW and environmental monitoring, where the rapid, on-site analysis of contaminants can guide immediate remediation efforts [18]. Recent portable MI-ECS prototypes have shown strong performances with high sensitivity and quick response times, making them useful in real-world applications [3].

These sensor systems’ wireless connectivity and portability enable the immediate transmission of data and allow for on-site analysis, which is vital for conducting timely environmental assessments. This capability not only enhances the efficiency of monitoring efforts but also supports rapid decision-making in response to environmental changes or pollution events [1,3,16,42].

## 3. Principles and Fabrication Techniques of MI-ECSs

### 3.1. Principles of MI-ECSs

MI-ECSs are an advanced class of sensors combining molecular imprinting technology (MIT) with electrochemical transduction to achieve high specificity and sensitivity in detecting target analytes [27,28]. The fundamental principle of these sensors involves establishing a recognition site within a polymer matrix that selectively binds to a target analyte [12,14]. This principle relies on MIPs, synthetic polymers that form molecular recognition sites through the polymerization of functional and crosslinking monomers around a template that mimics the target analyte(s) [9,11,12,13,14,22,26,27,28].

**Figure 10:**
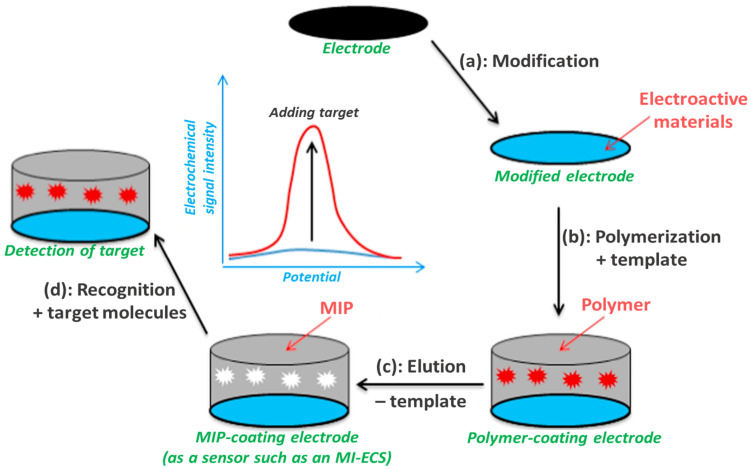
Schematic representation of the working principle of MI-ECS: fabrication and application. Reprinted from [64], with some modifications, Copyright (2018), with permission from Elsevier.

As illustrated in Figure 10, upon template removal, cavities that match the shape, size, and functional groups of the target molecule are left, allowing for selective binding and detection—the core principle of MIP systems [26,27,28]. The working principle underlying MI-ECSs involves making highly sensing-specific molecular recognition sites within the polymer matrix that interact with target molecules via non-covalent interactions, including hydrogen bonding, van der Waals forces, and electrostatic interactions [3,13,22,23,26]. An electrochemical transducer (ECT) linked to the MI-ECS system then converts the binding event into a measurable electrical signal, offering quantitative data on the presence and concentration of the target analyte [21,22,23,26].

Regarding any ECT methods, such as amperometric, potentiometric, and impedimetric methods, their selection and utilization in the MI-ECS system for WQM are based on criteria such as sensitivity, selectivity, response time, and operational range [14]. These methods should effectively detect target analytes at the low concentrations typical in environmental samples, like wastewater [19], and must distinguish the analyte from interferents commonly found in complex matrices, ensuring high specificity. The stability and reproducibility of the sensor’s responses are crucial for reliable monitoring. Additionally, the ECT method should be compatible with the sensor design and materials used, allowing for practical deployment in field conditions. Ultimately, the best choice aligns with the specific analyte’s chemical properties and monitoring requirements [41].

### 3.2. Fabrication Techniques of MI-ECSs

**Figure 11:**
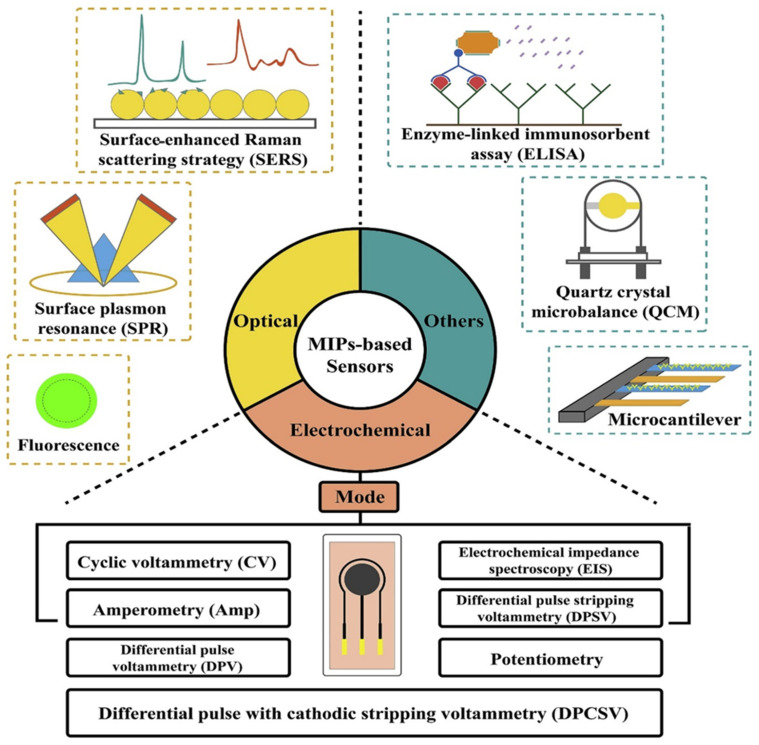
Schematic illustration of various techniques for developing MIP-based sensors. Reprinted from [65], Copyright (2019), with permission from Elsevier.

The fabrication techniques of MI-ECSs vary but generally include the preparation of the sensing matrix, template removal, and electrode modification by polymerization mechanisms [64], as illustrated in Figure 10. Generally, the polymerization techniques for developing MIP-based sensors can be initiated using either electrochemical (electropolymerization) or non-electrochemical (such as thermal, UV-induced (optical), and chemical polymerization) methods [65], as presented in Figure 6 and Figure 11, with electrochemical polymerization providing significant benefits based on its increased speed and enhanced control over polymer thickness at the nanometer scale [22,65]. In addition, the choice of monomers, crosslinkers, and solvents can significantly affect the sensor’s sensitivity and selectivity [65].

Regarding the electropolymerization process for developing MI-ECSs, as outlined in Section 3.1 and illustrated in Figure 10, after the polymerization and template removal, the electrodes are usually modified to enhance conductivity, often employing materials like graphene or carbon nanotubes. This modification increases the electroactive surface area and reduces the sensor’s response time [22]. Incorporating nanomaterials improves sensitivity, enabling the detection of low-concentration analytes [50,52], as applied in WQM.

MI-ECSs exhibit remarkable advantages, such as a high selectivity for target analytes, operational stability, and the capability for miniaturization, making them ideal for applications in WQM [9], food safety [65], environmental monitoring [49], and biomedical diagnostics [49]. Overall, the combination of molecular imprinting with electrochemical detection techniques holds great promise for developing next-generation sensors [13,14,49].

#### 3.2.1. Traditional Fabrication Techniques

Traditional fabrication techniques remain foundational in the development of MI-ECSs for WQM. Two notable methods—screen printing [66] and soft lithography [67]—offer distinct advantages for sensor construction and performance.

**Figure 12:**
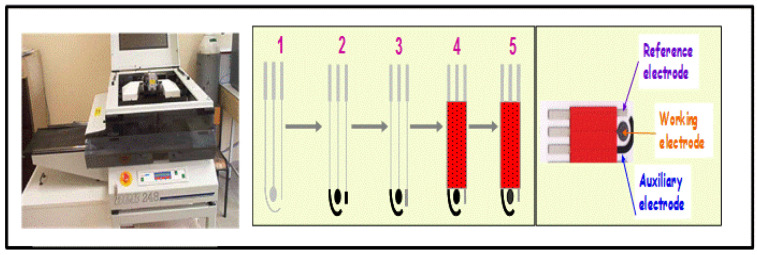
The fabrication of a three-electrode system: 1. a chemically inert substrate; 2. screen printing the WE and the CE; 3. screen printing the RE; 4. the screen printing of the protection paste; and 5. incubating the WE with the target analyte [68].

(i) Screen printing: This is a popular method for producing ECSs due to its simplicity, cost-effectiveness, portability, and suitability for mass production [66]. In brief, this method entails the application of a paste containing the MIP and conductive materials onto a substrate via a screen-printed electrode. The substrate is chemically inert and incorporates three electrodes: a WE, a RE, and a CE (or an AE), all of which are fabricated using screen-printing techniques. The fabrication of an electrochemical screen-printed sensor (ECS-PS) involves three main steps: producing the screen-printed electrode, designing its surface, and applying it for sensing, as illustrated in Figure 12.
  In a study carried out by Lin et al. [69], screen-printed carbon electrodes (SPCEs) were fabricated and modified using poly(3,4-ethylenedioxythiophene) (PEDOT) and PEDOT/MWCNTs, and their catalytic properties for nitrite detection were examined. The resulting MI-ECSs were successfully employed to measure nitrite concentrations in tap water samples. The resulting PEDOT- and PEDOT/MWCNTs-modified SPCE sensors showed distinct features, with low LODs of 1.72 and 0.96 μM, respectively, and high sensitivities of about 100 and 140 mAcm^−2^M^−1^, respectively, making them suitable for various contaminants in water. Screen-printed MI-ECSs enable the efficient integration of multiple electrodes on a single substrate, facilitating the simultaneous detection of multiple analytes. This scalability and applicability to different substrates make screen printing a favored choice for low-cost water monitoring solutions.
(ii) Soft lithography: This surface-imprinted fabrication process of MI-ECS preparation is conceptually simple but demands a careful preparation technique. The direct method involves fabricating a stamp from a self-assembled template array, which is pressed into a partially polymerized film. The stamp is held in place until polymerization is complete, after which it is removed, leaving behind binding sites on the surface as the template molecule is washed away [26]. In a report, Dickert et al. [70] developed highly crosslinked polymers with molecular cavities using molecular imprinting and soft lithography for surface-imprinted MI-ECSs. These sensors detect and monitor polycyclic aromatic hydrocarbons (PAHs) in drinking water at concentrations as low as several ng/L using fluorometric or mass-sensitive methods.

#### 3.2.2. Advanced Fabrication Techniques

Advancements in molecularly imprinted technology (MIT) for electrochemical sensing have significantly improved the development of novel MI-ECSs for WQM [50]. Notable methods include 3D printing [71], electrospinning [72], and chemical vapor deposition (CVD) [73], each offering unique advantages for sensor design and performance.

**Figure 13:**
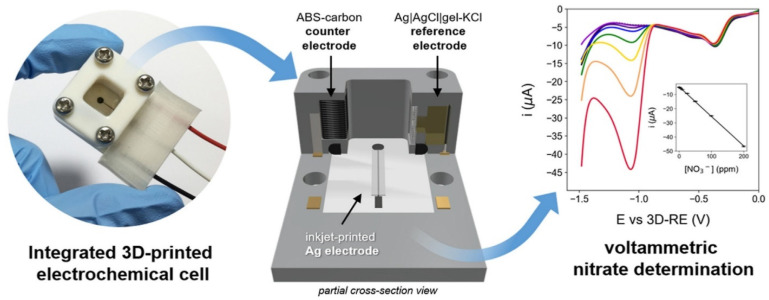
3D-printed MI-ECSs, based on inkjet-printed Ag electrode, for NO_3_^−^ detection. Reprinted from [71], Copyright (2021), with permission from Elsevier.

(i) 3D printing: This additive manufacturing technique facilitates the rapid prototyping of sensor components, allowing for customizable designs that can optimize fluid dynamics and sensor sensitivity [50,67]. 3D-printed MI-ECSs facilitate the inclusion of various sensing components and microfluidic pathways, thereby improving the detection of analytes in intricate water matrices. The accuracy inherent in 3D printing technology permits the creation of electrochemical cells that optimize the interaction between imprinted polymers and target analytes [50].
  Furthermore, 3D printing techniques have been effectively employed in fabricating pH sensors for WQM [74]. These 3D printing methodologies also offer significant benefits, including reduced costs and streamlined packaging solutions [63]. As illustrated in Figure 13, Sibug-Torres et al. [71] developed a 3D-printed ABS (acrylonitrile butadiene styrene)–carbon counter electrode (CE) and a modified Ag/AgCl/Gel-KCl reference electrode (RE) with a porous junction, enhanced by the electrodeposition of nanostructured Ag to increase the sensitivity for nitrate (NO_3_^−^) detection. Using linear sweep voltammetry (LSV), they detected NO_3_^−^ in synthetic brackish water samples at pH 8.0, achieving a sensitivity of 0.2086 μA ppm^−1^ and an LOD of 1.40 ppm.
(ii) Electrospinning: This technique generates micro/nanofibers with high surface *A*/*V* ratios, essential for optimal sensor performance [72]. Electrospun materials can serve as nanoscale scaffolds for molecular imprinting, resulting in enhanced mass transfer and improved sensitivity [75]. Incorporating electrospun fibers into MI-ECSs can significantly increase the effective binding sites accessible for target analytes, leading to lower detection limits and faster response times [72].
(iii) CVD: This fabrication method is essential for creating thin films of high-purity materials that can improve sensor robustness and electronic properties. Through CVD, uniform and conformal coatings can be achieved on electrode surfaces, enhancing the stability and reproducibility of MI-ECSs [73]. Thus, the CVD fabrication technique allows for the functionalization of the imprinted polymers, further improving specificity and selectivity for contaminants in water and environmental matrices [73].

#### 3.2.3. Advanced Surface Modification Strategies

Advanced surface modification strategies are pivotal in enhancing the performance of MI-ECSs for WQM. Specifically, functionalization with nanomaterials [52] and the incorporation of bio-recognition elements, like enzymes, antibodies, and aptamers, are noteworthy approaches [65,73].

(i) Functionalization with nanomaterials: As discussed previously, the integration of nanomaterials, such as GR, CNTs, and metallic nanoparticles (MNPs), into MI-ECSs significantly enhances electrical conductivity, surface area, and electrocatalytic activity [52]. For instance, graphene oxide (GO) can be incorporated into the imprinted polymer matrix, improving electron transfer and the sensitivity to target analytes [49,52]. The unique properties of nanomaterials facilitate a higher concentration of binding sites, resulting in improved LODs and faster response times. Moreover, these materials can stabilize the imprinted polymer, extending sensor longevity and stability in varying environmental conditions [52].
(ii) Bio-recognition elements: The incorporation of bio-recognition elements, such as enzymes, antibodies, and aptamers, into MI-ECSs enhances selectivity and specificity for target contaminants [65]. Enzymes can catalyze reactions specific to the contaminants, allowing for direct quantification through electrochemical signals [76]. Antibodies provide high specificity due to their unique binding capabilities [77], while aptamers offer the advantages of chemical stability and the ease of synthesis [78], making them effective for sensing applications in complex matrices. These bio-recognition strategies enable the sensors to effectively differentiate between similar molecules, crucial for accurate water quality assessments.

## 4. Applications of MI-ECSs in WQM

Molecularly imprinted electrochemical sensors (MI-ECSs) have become essential instruments in water quality management (WQM) due to their ability to selectively and sensitively detect a wide range of contaminants in water systems. These sensors can be tailored to target specific contaminants, enhancing their effectiveness in WQM. One of the key advantages of MI-ECSs is their capability to provide real-time, on-site analysis, which significantly improves response times in environmental monitoring. This rapid detection enables quicker compliance with safety standards and supports timely remediation efforts when water issues arise. Consequently, MI-ECSs play a vital role in maintaining and managing water quality (WQ) across various settings, as illustrated in Table 2. MI-ECSs are utilized for detecting a wide range of contaminants, including organic, inorganic, and biological contaminants (like toxins, pathogens, enzymes, and hormones) and other emerging contaminants and toxicants (ECs and Ts), as detailed in this section.

### 4.1. MI-ECSs for Detecting Organic and Inorganic Contaminants in Water Systems

As in previous discussions in this review paper, MI-ECSs have become crucial in WQM for their capability to detect specific organic and inorganic contaminants in water systems selectively. These sensors utilize MITs to create imprinted sites that bind target analytes, allowing for high sensitivity and specificity in detecting contaminants, such as inorganics (mainly HM ions) [34], agrochemicals (like pesticides) [13,33], pharmaceuticals [10,122], cosmetics or personal care products (PCPs) [13], endocrine disruptive chemicals (EDCs) [13], and other organic compounds [13,33], as presented in Table 2. For example, MI-ECSs have been effectively employed to monitor contaminants like nitrates and phosphates, which are crucial parameters for assessing eutrophication and overall water quality [22,33].

Furthermore, the integration of MI-ECS technology into portable devices facilitates real-time monitoring, enabling the timely assessment of water contamination and aiding in the management of water resources [17,22,65]. This innovative approach promises improved environmental protection and a safer water supply across various contexts.

### 4.2. MI-ECSs for Detecting Pathogenic Contaminants in Water Systems

**Figure 14:**
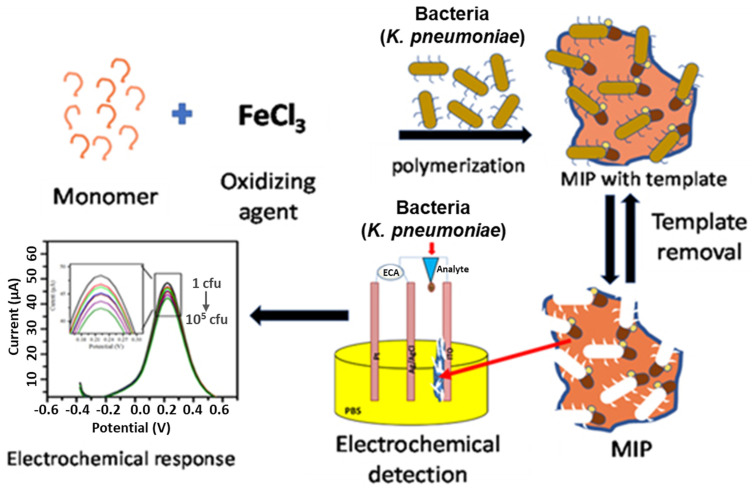
Schematic illustration (with some modifications) of the MIP/ITO electrode formation and application for sensing *K. pneumoniae* [105].

The progress in technology and the principles underlying the use of MI-ECSs as biosensors and biomarkers represents a significant improvement over traditional methods, such as the enzyme-linked immunosorbent assay (ELISA) and the real-time polymerase chain reaction (RT-PCR) [123]. This advancement has facilitated the detection of pathogens (including bacteria, viruses, and fungi), various toxins, antibodies, and enzymes in water bodies by utilizing a receptor, a transducer, and a recognition element to identify the target molecule in question. For instance, Babamiri et al. [111] successfully utilized an MIP-based electrochemiluminescence (MIP-ECL) sensor as an MI-ECS for the sensitive and selective detection of the human immunodeficiency virus type 1 (HIV-1) gene in aqueous environments, employing Europium sulfide nanocrystals (ESNCs) as the signal transducer. In another study, Doostmohammadi et al. [124] developed monodisperse, MIP-based, core-shell microparticles (MIP-MPs) that serve as biorecognition elements with a strong binding affinity for a bacterial surrogate, *E. coli OP50*. In addition, Sharma et al. [105] developed a novel MIP/ITO electrode using interfacial oxidative polymerization and electrophoretic deposition (Figure 14). This electrode was designed to detect *Klebsiella pneumoniae* in spiked urine samples due to the pathogen’s potential to cause infectious diseases and contaminate water systems if not properly detected, monitored, and controlled. The sensor was found to be highly selective and sensitive to *K. pneumoniae* with an LOD of 1.352 CFU/mL.

**Figure 15:**
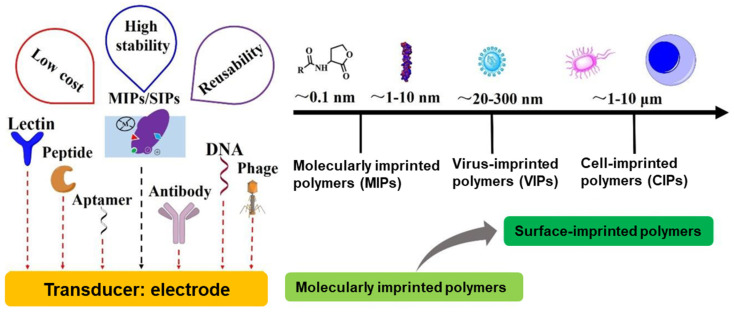
Electrochemical biosensors as biomarkers with various receptors for detecting pathogens based on particle sizes [125].

As illustrated in Figure 15, biosensors consist of receptors that specifically recognize analytes by binding to them and transducers that convert this interaction into measurable signals, often using electrodes in electrochemical biosensors. Natural receptors, such as antibodies, aptamers, peptides, nucleic acids (DNA and RNA), phages, and lectins are used as receptors for biosensing in conventional biosensors. Despite their high selectivity, these sensors face challenges with durability and stability under extreme conditions [125]. These challenges are overcome by MIPs, and surface imprinted polymers (SIPs) serve as robust artificial receptors with cost-effectiveness, enhancing the performance of electrochemical biosensors, especially in the context of infectious disease detection [126].

Table 2 presents some applications of MI-ECSs for detecting pathogenic contaminants in water bodies. The application of MI-ECSs in biosensing and biomarker analysis and generally for WQM offers numerous advantages, including high stability, remarkable selectivity, rapid results, cost-effectiveness, and high accuracy, as compared to other sensor types (Table 3).

### 4.3. MI-ECSs for Detecting Other Emerging Contaminants and Toxicants in Water Systems

The application of MI-ECSs has also expanded to include the detection of a wide range of other ECs and Ts in water systems and other environmental media, as presented in Table 2. These chemicals include cosmetic products or PCPs and their residues, hormonal compounds, endocrine-disrupting substances (EDSs), pharmaceuticals, dyes, etc., and are often found in low or trace concentrations in the range of picomolar (pM) to nanomolar (nM) [127]. They are typically characterized by their persistence, toxicity, and tendency to bioaccumulate, making their presence in the environment a concern. Their trace levels usually pose a significant challenge for detection in complex matrices, causing the need for highly sensitive, selective, effective, specific, reproducible, and accurate sensors, such as MI-ECSs, with lower detection limits for their real-time analysis and monitoring.

**Figure 16:**
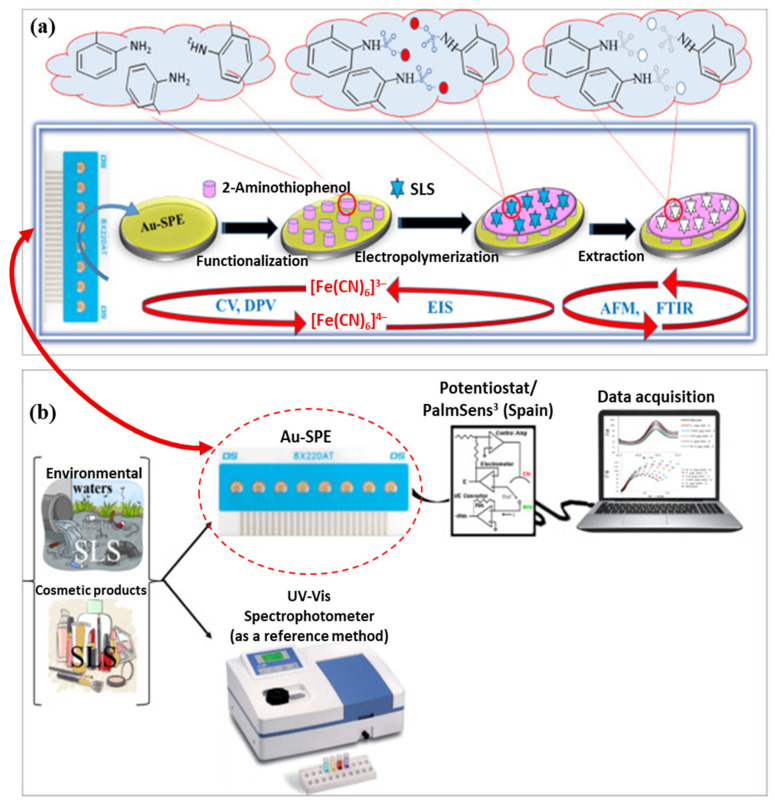
Schematic illustrations of (**a**) the formation and mechanism of action of the MIP-based Au-SPE (MIP@Au-SPE) sensor fabricated via electrochemical polymerization and (**b**) the portable device system of the MIP@Au-SPE sensor and the UV-Vis spectrophotometer employed for electrochemical detections and measurements of sodium lauryl sulfate (SLS) in environmental water and cosmetic product samples. (AFM: atomic force microscopy; FTIR: Fourier transform infrared spectroscopy). Reprinted from [119], with some modifications, Copyright (2018), with permission from Elsevier.

For example, in a study conducted by Motia et al. [119], an MIP-based sensor (MIP@Au-SPE) was developed by the electrochemical polymerization of 2-aminothiophenol (2-ATP) onto a screen-printed gold electrode (Au-SPE), as illustrated in Figure 16. Sodium lauryl sulfate (SLS) was used as the template molecule to create binding sites specific to SLS. This sensor was designed to detect and measure SLS levels in both environmental water and cosmetic product samples, with a UV-Vis spectrophotometer serving as the reference method. The sensor demonstrated good linearity for SLS binding within the concentration range of 0.1–1000 pg/mL and with an LOD of 0.18 pg/mL. Furthermore, the sensor exhibited high selectivity, reproducibility, and stability, suggesting its potential for use in environmental analysis, including WQM.

## 5. Comparison of MI-ECSs with Other Existing Technologies for WQM

As presented in Table 3, MI-ECSs stand out in WQM due to their impressive selectivity, affordability, and durability compared to other methods, like traditional ECs (TECs), conventional biosensors, optical sensors, and remote sensing technologies. They employ unique MIPs that are specifically designed for target analytes, enhancing their detection accuracy and effectiveness. However, their intricate fabrication processes and susceptibility to interference are notable limitations that must be evaluated based on the specific requirements of monitoring applications [12,13,17].

In contrast, TECs and biosensors also offer their advantages in WQM, such as rapid response times and heightened sensitivity, respectively. TECs provide portable solutions but may face challenges with selectivity, while biosensors excel in specificity but can be costly and less stable. Optical sensors, on the other hand, are effective for detecting low concentrations through light absorption but are sensitive to environmental changes [16,63]. In addition, remote sensing technology monitors water quality via satellites or aerial sensors by analyzing spectral data for parameters like TSSs, chlorophylls, oil spills, and cyanobacteria with broad spatial coverage and rapid data acquisition. However, remote sensing accuracy is affected by atmospheric interference, resolution limits, limited depth penetration, and data interpretation issues, necessitating validation with direct water samples.

Ultimately, choosing the most suitable WQM technology relies on a careful assessment of the specific monitoring tasks and criteria to be met. Each technology exhibits unique strengths and weaknesses, making it essential to match the method to the monitoring context and environmental conditions for optimal results [31].

## 6. Challenges and Future Research Directions

The functionalization and modification of electrochemical sensors (ECSs) with MIPs show significant promise when combined with various nanomaterials and some other innovative fabrication strategies to create sensitive and selective MI-ECSs. They provide benefits such as low cost, reusability, and biomimetic features that outperform natural receptors like antibodies and enzymes. Recent advancements in nano-sized MIPs harness the electrical conductivity and catalytic properties of nanomaterials, coupled with MIPs’ high selectivity, enhancing their effectiveness in various electrochemical sensing applications. However, several challenges must be addressed to integrate this technology into everyday life. These challenges include issues related to its cost and scalability, selectivity, regeneration, stability and lifespan, limited detection range, reproducibility, signal variability, calibration complexity, data interpretation, and field deployment. They extend beyond water quality and environmental monitoring issues as they also affect various applications of MI-ECSs, including food safety and electronics industries.

Addressing these issues will significantly enhance the effectiveness of MI-ECSs in WQM. For example, integrating nanomaterials into the fabrication of MI-ECSs has proven to be an innovative solution that can help overcome several of these obstacles. Some other innovative and efficient strategies for addressing various challenges associated with MI-ECSs in WQM are presented below.

(i) The development of robust MI-ECSs: there is a need for developing stable, user-friendly sensors for various applications in water, food, environmental, and clinical analyses.
(ii) The design and fabrication of portable MI-ECSs: creating compact, wearable, and implantable devices that can analyze multiple components simultaneously is challenging due to the requirements for cost reduction, size minimization, and lower energy consumption.
(iii) Detection across concentrations: producing electrochemical sensors capable of detecting analytes at extremely low concentrations (femto molar (fM) or atto molar (aM)) with stability remains difficult.
(iv) The use of non-toxic nanomaterials: the incorporation of safe, novel nanomaterials in MI-ECSs is essential for enhancing safety and performance.
(v) Electrode contamination: surface contamination can lead to electrode fouling or poisoning, affecting voltammetric responses and causing inter-laboratory variability.
(vi) Signal stability and repeatability: maintaining consistent sensor performance is particularly challenging when analyzing real environmental samples.
(vii) Temperature and location limitations: sensors’ performance can be restricted in extreme conditions, impacting their usability in remote locations.
(viii) Paper-based electrodes: while paper-based MI-ECSs offer low-cost options for heavy metal detection, their stability, reproducibility, and sensitivity need improvement for field applications.
(ix) Advancements in electronics incorporation: developing electronics and microfabrication techniques is necessary to incorporate microchips into MI-ECSs for the continuous monitoring of water and environmental samples.
(x) The detection of biomacromolecules: although MI-ECSs excel at detecting small analytes, there is still a significant need for effective sensors for larger molecules, such as proteins and bacterial cells.
(xi) The exploration of more effective functional monomers and nanopatterned electrodes: exploring more functional monomers and using nanopatterned electrodes can enhance chemical recognition by promoting the formation of MI-ECSs with better cavity effectiveness.
(xii) Biosensor improvement with machine learning integration: machine learning (ML) and artificial intelligence (AI) can significantly enhance the design and recognition features of MI-ECSs, signaling advancements in MIP-based ECS technology for biosensing and chemosensing in WQM applications.
(xiii) Addressing data interpretation challenges (such as signal variability, background noise, interferences, and matrix effects): these challenges can be minimized by employing robust calibration protocols, utilizing advanced data analysis techniques, such as ML, and implementing standard reference materials to enhance data accuracy and reliability in real-world applications.

**Figure 17:**
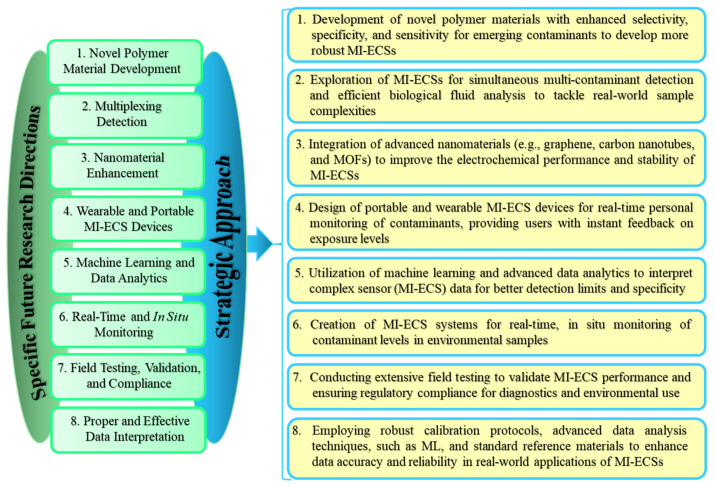
Specific future research directions and a strategic approach to MI-ECS technology for detecting ECs and Ts across various matrices, based on the real-time monitoring capabilities of MI-ECSs.

In summary, to develop effective MI-ECSs for emerging contaminants and real-time monitoring, Figure 17 presents the key areas that should be prioritized.

## 7. Conclusions

The use of MI-ECSs for WQM and in other applications based on molecularly imprinted technology (MIT) and electrochemistry is growing rapidly, with their fabrication in various strategic innovations involving the use of nano-structured materials, microfluidics, screen printing, and 3D printing, among others. The design, architecture, principles, and fabrication of MI-ECSs reported in this review have shown promising results that demonstrate the applicability of this novel electrochemical sensing strategy for detecting diverse analytes in water systems and complex matrices, such as food and environmental samples, pharmaceuticals, PCPs, and biological fluids, among others. The advancement of MI-ECSs in targeting biomacromolecules (such as proteins, nucleic acids, polysaccharides, and whole cells) and microorganisms (such as bacteria, viruses, and micromycetes) is still in its early stages. However, recent developments in biomimetic and magnetic MI-ECSs indicate that MIPs, when combined with nanomaterials and innovative fabrication techniques, can match biological receptors in regard to their sensitivity, selectivity, stability, and ease of synthesis. This progress suggests that MI-ECSs have the potential for widespread applications in real-time WQM, as well as environmental monitoring.

## Figures and Tables

**Table 1:** Types, principles, benefits, and limitations of EC sensing techniques employed in MI-ECS systems [14,40,41].

EC Sensing Technique	Principle	Benefits	Limitations
Amperometry	Measures the current at a fixed potential, related to the analyte concentration.	– High sensitivity. – Real-time monitoring.	– Limited selectivity. – Possible interference.
CV	Measures the current while sweeping potential linearly between two limits.	Provides information on redox reactions.	– Requires detailed analysis. – Not highly selective.
SWV	Superimposes square waves on a staircase potential to measure the current.	– High sensitivity. – Fast scan rates.	Requires complex instrumentation.
DPV	Superimposes small pulses on a linear potential sweep, measuring the current difference.	– High sensitivity. – Low detection limits.	– Complex data analysis. – Slow.
LSV	Measures the current during a linear potential sweep in one direction.	– Simpler than the CV method. – Useful for reaction kinetics.	– Less information than the CV method. – Possible drift.
SV	The preconcentration step is followed by a voltammetric measurement.	– Extremely sensitive. – Useful for trace analysis.	– Time-consuming. – Requires careful optimization.
EIS	Measures impedance as a function of frequency.	– Easy integration with other sensing techniques. – Real-time monitoring.	– Complex data interpretation. – Time-consuming.
Potentiometry	Measures the difference in the potential between electrodes at zero current.	– Simple, selective, and inexpensive.	– Limited to specific ion detection.
Conductometry	Measures the conductivity of a solution.	– Simple and fast. – No reference electrode is needed.	– Low specificity. – Affected by temperature changes.

**Table 2:** Some applications of MI-ECSs in WQM.

MI-ECS Detection Technique	Sample Matrix	Target Analyte(s)	Template, Functional Monomer(s), Initiator, and Crosslinker	Sensor Fabrication	Mode and the Probe	Linear Range (LR)	LOD	Ref.
Applications in detecting organic contaminants								
CV	Tap water	Dibutyl phthalate	Dibutyl phthalate-template, MAA, AIBN, EGDMA	Coating of carboxylate-SiO_2_ with MIP and its dropping on AuNPs/MWCNTs/GCE	Indirect, [Fe(CN)_6_]^3−^	10^−7^–10^−2^ g/L ^a^	5.09 ng/L	[79]
	Tap water	*p*-Nitrophenol (*p*-NP)	*p*-NP-template, aniline, APS, *NR*	Coating of GO/CPE with MIP-PANI	Direct	60–140 μM	20 μM	[80]
	Tap water	Amoxicillin (AMO) antibiotic	AMO-template, MAAM, TEOS, VTMOS	Hybrid organic–inorganic MIP film (AMO-MIP) was integrated with an SPR sensor	Indirect, [Fe(CN)_6_]^3−/4−^	0.1–2.6 nM	73 pM	[81]
DPV	Tap water	Perfluorooctane sulfonate Genistein	PFOS-template, *o*-PD, *NR*, *NR* Genistein-template, *o*-PD, *NR*, *NR*	Au nanostar/GCE was coated with MIP	Indirect, FcCOOH	0.05–5.0 nM	0.015 nM	[82]
	Tap and sea waters	Bisphenol A (BPA)	BPA-template, MIPPy, *NR*, *NR*	MIPPy/GQDs/GCE fabrication	Indirect, [Fe(CN)_6_]^3−^	0.1–50 μM	0.04 μM	[83]
	Tap, river, and sea waters	RDX explosive	RDX-template, MAA, AIBN, EGDMA	Drop-coating of MIP/MWCNTs mixture on GCE	Direct	0.1–10.0 nM and 0.01–1.00 µM	20 pM	[84]
Amp and CAmp	Tap and mineral waters	BPA	BPA-template, MMA, EGDMA	MIP synthesized on Fe_3_O_4_ NPs (a form of SPIONs) and spin-coated onto SPE as core-shell NPs	Direct	0.01–3.0 μM	20.53 nM	[85]
SWV	Groundwater	Isoproturon	Isoproturon-template, Py, *NR*, *NR*	CV electropolymerization of GCE with Py	Indirect, ferrocene	0–0.5 μM	2.2 μg/L	[86]
Potentiometry	Sea, ground, and tap waters	γ-HCCH (pesticide)	γ-HCCH-template, MAA, AIBN, EGDMA	MWCNT-MIP film on the surface of the Cu electrode	Direct	1.0 nM–1.0 mM	0.1 nM	[87]
Conductometry	Environmental waters	PAHs	Anthracene-template, BPA + MDI, *NR*, phloroglucinol	Screen-printed interdigital Au electrodes on glass coated with MIP polyurethane layers	Direct	*NR*	1.3 nM	[88]
EIS	Wastewater	Methidathion (insecticide)	Methidathion-template, MBAA, AIBN, EGDMA	Drop casting of MIP@sol-gel/PEG on the surface of SPCE	Indirect, [Fe(CN)_6_]^3−/4−^	40–200 μg/L	5.14 μg/L	[89]
DV	Plastic-bottled drinking water	BPA	BPA-template, *NR*, *NR*, *NR*	MIP/CHI/GR/ABPE (MIP-CHI-GR composite film)	*NR*	8.0 nM–20 μM	6.0 nM	[90]
CV, DPV	River water	Chlorpyrifos (CPF)	CPF-template, MAA, *NR*, EGDMA	MIP/GCE fabrication	Indirect, [Fe(CN)_6_]^3−/4−^	0.1 nM–10.0 μM	4.08 nM	[91]
	Lake water	DCP	DCP-template, MAA, AIBN, EGDMA	MIP/GO/GCE fabrication	Direct	0.004–10.0 μM	0.5 nM	[92]
	Tap, rain, and lake waters	TBBPA	TBBPA-template, *NR*, *NR*, *NR*	CV electrodeposited TBBPA imprinted film on Ni/GP/CE, forming MIP/Ni/GP/CE	Indirect, [Fe(CN)_6_]^3−/4−^	0.5 nM–10.0 μM	0.13 nM	[93]
CV, DPV, EIS	Waste and mineral waters	Triclosan (TCS)	TCS-template, AAM, APS, MBAA	ECS based on Au-SPE/PVC-COOH/MIP	Indirect, [Fe(CN)_6_]^3−/4−^	0.1–1000 ng/L	0.23 ng/L	[19]
	River, spring, and tap waters	Atorvastatin (ATV) drug	ATV-template, 4-ABA, *NR*, *NR*	CV electropolymerization of SPCE with 4-ABA	Indirect, [Fe(CN)_6_]^3−/4−^	0.05–2.0 mM	0.049 mM	[94]
SERS	River water	Caffeine	Theophylline-template, MAA, *NR*, EGDMA	Single-step AgNPs@MI@S-PE-SERS nanosensors	Direct	0–100 μg/L	100 ng/L	[95]
Applications in detecting inorganic contaminants								
CV	Tap water	Nitrite (NO_2_^−^)	*NR*	PEDOT/MWCNTs-modified SPCE via electropolymerization	*NR*	0.05–1 mM	0.96 μM	[69]
	Ground and waste waters	Hg^2+^	Hg^2+^-THPP-template, MAA, AIBN, EGDMA	MWCNTs/GCE via thermal precipitation polymerization	*NR*	0.01–700 µM	5.0 nM	[96]
DPV	Commercial drinking water	Cu^2+^	Cu^2+^-template, *p*-PD, *NR*, *NR*	Cu^2+^-MIP film (SPPtE) sensor via electropolymerization	*NR*	0.95–244 nM	2.7 nM	[97]
CV, DPV	Tap and river waters	Hg^2+^	Hg^2+^-template, GQDTU, AIBN, EGDMA	GQDTU/MIP/GCE fabricated by suspension polymerization	*NR*	CV: 60 nM–0.85 µM DPV: 50 nM–23 µM	CV: 30.2 nM DPV: 23.5 nM	[98]
	Yellow River water	Pb(II) (or Pb^2+^)	Pb(II) ions-template, *NR*, *NR*, *NR*	Fabrication of imprinted SAMs on AuES	Indirect, [Fe(CN)_6_]^3−^	0.3–50 μM	0.2 μM	[99]
	Tap water	As(V) ions (as arsenate salt)	*Depending on the polymer types*	AAGO-PDDA-PA/GCE fabricated by copolymerization	Indirect, [Fe(CN)_6_]^3−/4−^	≤30 µM	8.99 ppb	[100]
DPASV	Mineral, river, and tap waters	Cd(II) (or Cd^2+^)	Cd(II) ions-template, 4ʹ-VPTP, *NR*, EGDMA	Cd-MIP/CPE fabricated by bulk copolymerization	*NR*	4.0 nM–0.5 µM	1.94 nM	[101]
Potentiometry	Spiked river, tap, and dam waters	Cu^2+^	Cu^2+^-template, MTMA, AIBN, EGDMA	GO/CNTs/Cu^2+^-MIP fabrication	Direct	1.0 µM–0.1 M	0.4 µM	[102]
Applications in detecting pathogenic contaminants								
CV, DPV	Human serum	HIV-p24	HIV-p24-template, AAM, APS, MBA	MWCNT-modified GCE by surface polymerization	GCE	0.0001–2 ng/mL	0.083 pg/mL	[103]
	Cell samples	*S. epidermidis*	*S. epidermidis*-template, 3-APBA, *NR*, *NR*	3-APBA electropolymerization with Au electrode	Indirect, [Fe(CN)_6_]^3−/4−^	10^3^–10^7^ CFU/mL	*NR*	[104]
	Spiked urine samples	*K. pneumoniae*	*K. pneumoniae*-template, Py, *NR*, *NR*	MIP/ITO electrode fabricated via interfacial oxidative polymerization	Indirect, [Fe(CN)_6_]^3−/4−^	1.0–10^5^ CFU/mL	1.352 CFU/mL	[105]
	Spiked food samples	Mycotoxins (AFB1) and FuB1)	AFB1-template or FuB1-template, aniline, APS, *NR*	MIP-A/ITO and MIP-F/ITO glass electrodes fabricated via chemical oxidative polymerization	Indirect, [Fe(CN)_6_]^3−/4−^	1.0 pg/mL–500 ng/mL	AFB1: 0.313 pg/mL FuB1: 0.322 pg/mL	[106]
CV, CA	Food and water solutions	*S. cerevisiae*	*S. cerevisiae*-template, Py, *NR*, *NR*	MIP-modified SPCE by electropolymerization	*NR*	10^2^–10^7^ CFU/mL	10^1.12^–10^1.25^ CFU/mL	[107]
CV, EIS	Milk solutions	*S. aureus*	*S. aureus*-template, TAA, *NR*, *NR*	BICP film-based impedimetric sensor by electropolymerization	Indirect, [Fe(CN)_6_]^3−/4−^	10–10^8^ CFU/mL	2 CFU/mL	[108]
	Saliva solution	SARS-CoV-2-RBD	SARS-CoV-2-RBD-template, *o*-PD, *NR*, *NR*	MIP/MP-Au-SPE fabricated via electropolymerization of *o*-PD	Indirect, [Fe(CN)_6_]^3−/4−^	2.0–40.0 pg/mL	0.7 pg/mL	[109]
CV, SWV	Apple juice solutions	Patulin (Pat) toxin	Pat-template, [EMIM][BF_4_], AIBN, EGDMA	MIP/Fe_3_O_4_/GO/GCE via imprinting polymerization	Indirect, [Fe(CN)_6_]^3−/4−^	0.001 nM–0.25 µM	0.333 pM	[110]
ECL, CV	Serum sample	HIV-1 gene	HIV aptamer-template, *o*-PD, *NR*, *NR*	Direct electropolymerization of *o*-PD	Indirect, [Fe(CN)_6_]^3−/4−^	3.0 fM–0.3 nM	0.3 fM	[111]
CV, DPV, EIS	Environmental waters	MC-LR (from algal blooms)	L-arginine, *p*-ATP + MAA, *NR*, *NR*	MIP/A@M/G/G made via in situ electrochemical polymerization	Indirect, [Fe(CN)_6_]^3−/4−^	0.08–2.0 μg/L	0.0027 μg/L	[112]
Applications in detecting some other emerging contaminants and toxicants								
CV	Spiked water	17*β*-E_2_ (oestrogen)	17*β*-E_2_-template, 4-aminothiophenol, *NR*, *NR*	Nanowell-based MI-ECS with Au-film-coated electrode via Ag/Au alloy dissolution	Indirect, [Fe(CN)_6_]^3−/4−^	1.0 pM–10.0 μM	0.1 pM	[113]
	Tap water, river water, and soils	SMZ, PL, AC, TL	SMZ-template, *o*-ATP, *NR*, *NR*	MIP/ATP@AuNPs fabricated via electrochemical polymerization	Indirect, [Fe(CN)_6_]^3−/4−^	0.03–140 μM	0.013 μM	[114]
CV, EIS	Biological fluids	Testosterone	Testosterone-template, *o*-PD, *NR*, *NR*	MIP/GO modified electrode via electropolymerization	Indirect, [Fe(CN)_6_]^3−/4−^	1.0 fM–1.0 µm	0.4 fM	[115]
	Serum samples (or any clinical samples)	FSH	FSH-template, MMA, *NR*, EGDMA	NiCo_2_O_4_/rGO/MIP-modified ITO electrode fabricated via electrochemical polymerization	Indirect, [Fe(CN)_6_]^3−/4−^	0.1 pM–1.0 µM	0.1 pM	[116]
	River water	17*β*-E_2_ (oestrogen)	17*β*-E_2_-template, oleic acid, APS, EGDMA	MIP/Fe_3_O_4_/SPCE via m-NPs imprinting polymerization	Indirect, [Fe(CN)_6_]^3−/4−^	0.05–10 μM	20 nM	[117]
	Environmental water	Erythromycin (Ery)	Ery-template, *m*-PD, *NR*, *NR*	MIP/m-PD/SPE fabricated via electrochemical polymerization	Indirect, [Fe(CN)_6_]^3−/4−^	*NR*	0.1 nM	[66]
CV, DPV	Environmental matrices and foods	TC, OTC, AMX, CAP	TC-template, Py, *NR*, *NR*	MIOPPy-AuNP/SPCE fabricated by electropolymerization	*NR*	1–20 μM	0.65 μM	[118]
CV, DPV, EIS	Environmental waters and cosmetics (PCPs)	SLS	SLS-template, *o*-ATP, *NR*, *NR*	MIP-based Au-SPE (MIP@Au-SPE) fabricated via electrochemical polymerization	Indirect, [Fe(CN)_6_]^3−/4−^	0.1–1000 pg/mL	0.18 pg/mL	[119]
	Environmental matrices and foods	Tartrazine (Tz)	Tz-template, L-arginine, *NR*, *NR*	ZnO/MIP@P-Arg/CPE fabricated via electrochemical polymerization	Indirect, [Fe(CN)_6_]^3−/4−^	8.0–112.0 nM and 0.25–5.0 µM	2.7 nM	[120]
CV, SWAdASV	Environmental matrices, foods, and medicines	SY dye	SY-template, MSA, AIBN, EGDMA	Fe_3_O_4_@MIP/CPE (MMIPs) fabricated via precipitation polymerization	*NR*	1.51 μM–1.51 mM	86.242 μM	[121]

^a^ Linear response in a semi-log scale. *NR: not reported*; CFU: colony forming unit; LOD: limit of detection; AuNPs: gold nanoparticles; 17*β*-E_2_: 17*β*-estradiol; Amp: amperometry; CAmp: chronoamperometry; PFOS: perfluorooctanoic acid potassium salt; *o*-PD: *ortho*-phenylenediamine; *p*-PD: *para*-phenylenediamine; FcCOOH: ferrocene carboxylic acid; NPs: nanoparticles; Py: pyrrole; MIPPy: molecularly imprinted polypyrrole; GQDs: graphene quantum dots; CPE: carbon paste electrode; SPE: screen-printed electrode; SPIONs: superparamagnetic iron oxide nanoparticles; DCP: 2,4-dichlorophenol; DV: derivative voltammetry; ABPE: acetylene black paste electrode; CHI: chitosan; APS: ammonium persulphate; RDX: “Royal Demolition eXplosive” or “Research Department eXplosive” (such as trinitroperhydro-1,3,5-triazine); PEG: poly(ethylene glycol); MBA: *N*,*Nʹ*-methylene bisacrylamide; TEOS: tetraethoxysilane; VTMOS: vinyltrimethoxysilane; MAAM: methacrylamide; MI@S-PE: molecularly imprinted coupled with solid-phase extraction (S-PE); SERS: surface enhanced Raman scattering; TBBPA: 3,3ʹ,5,5ʹ-tetrabromobisphenol A; Ni/GP/CE: nickel nanoparticles-graphene modified carbon electrode; PVC-COOH: carboxylic polyvinyl chloride; AAM: acrylamide; γ-HCCH: gamma-hexachlorocyclohexane (lindane); MDI: 4,4ʹ-methylenediphenyldiisocyanate; *S. epidermidis*: *Staphylococcus epidermidis*; *S. aureus*: *Staphylococcus aureus*; 3-APBA: 3-aminophenylboronic acid; PC: polycarbonate; PTMS: phenyltrimethoxysilane; MTMS: methyltrimethoxysilane; DPASV: differential pulse anodic stripping voltammetry; 4ʹ-VPTP: 4ʹ-(4-vinylphenyl)- 2,2ʹ:6ʹ,2ʹ’-terpyridine; THPP: 5,10,15,20-tetrakis(3-hydroxyphenyl)-porphyrin (as a ligand); GQDTU: graphene quantum dot (GQD) functionalized with thiourea (TU) derivative; SAMs: self-assembled monolayers; AuES: gold electrode surface; SPPtE: screen-printed platinum electrode; MTMA: 5-methyl-2-thiozylmethacrylamide; AAGO-PDDA-PA/GCE: polyaniline (PA) and poly(diallyl dimethyl ammonium chloride) (PDDA) copolymer doped with acrylic acid-functionalized graphene oxide (AAGO) imprinted glassy carbon electrode (GCE); BICP: bacteria-imprinted conductive poly(3-thiopheneacetic acid); TAA: 3-thiopheneacetic acid; [EMIM][BF_4_]: 1-ethyl- 3-methylimidazolium tetrafluoroborate; CA: chronoamperometry; *S. cerevisiae*: *Saccharomyces cerevisiae*; SARS-CoV-2-RBD: severe acute respiratory syndrome coronavirus 2 receptor-binding domain; MP-Au-SPE: macroporous gold screen-printed electrode; m-NPs: magnetite nanoparticles; *m*-PD: *meta*-phenylenediamine; TC: tetracycline; OTC: oxytetracycline; AMX: amoxicillin; CAP: chloramphenicol; MIOPPy: molecularly imprinted overoxidized polypyrrole; *o*-ATP (or 2-ATP): *ortho*-aminothiophenol (or 2-aminothiophenol); SMZ: simazine; PL: picloram; AC: acetochlor; TL: terbuthylazine; SLS: sodium lauryl sulfate; FSH: follicle-stimulating hormone; ITO: indium tin oxide; P-Arg: poly-arginine; SY dye: Sunset Yellow dye; MSA: methylene succinic acid; SWAdASV: square wave adsorptive anodic stripping voltammetry; *K. pneumoniae*: *Klebsiella pneumoniae*; AFB1: aflatoxin B1; FuB1: fumonisin B1; MIP/A@M/G/G: MIP/AuNPs@MWCNTs/GQDs/GCE; *p*-ATP (or 4-ATP): *para*-aminothiophenol (or 4-aminothiophenol); MC-LR: microcystins with leucine arginine.

**Table 3:** Comparison of ECSs with other existing technologies for WQM.

Method	Principle	Advantages	Limitations
MI-ECSs	MI-ECSs feature MIPs that are crafted with precise template molecules, allowing the MIPs to retain complementary binding sites after the removal of the template, which mimics the target analyte’s shape and functional groups (FGs).	Highly sensitive, selective, effective, specific, reproducible, versatile, cost-effective, stable, portable, and accurate for real-time and on-site analysis with faster response times, lower detection limits, and higher potential for miniaturization.	Complex fabrication, interference issues.
2. Traditional ECs (TECs)	They measure changes in current or voltage in response to the redox reactions of target analytes in an electrochemical cell.	Fast response, portable.	Limited selectivity, signal drift.
3. Conventional biosensors (like ELISA and RT-PCR)	These are enzyme-based and antibody-based sensors that utilize specific biological interactions, mainly antibody–antigen and enzyme–substrate interactions, to detect target analytes based on coupling with a transducer for signal output.	High sensitivity and specificity, ability to use diverse detection mechanisms, such as optical and electrochemical mechanisms.	Costly, stability issues.
4. Optical sensors (like UV-Vis spectrophotometry and fluorescence sensors)	Optical sensors rely on light absorption or emission properties of analytes by measuring changes in light intensity or wavelength.	High sensitivity, non-destructive.	Environmental sensitivity, equipment cost.
5. Remote sensing technologies (RSTs)	RSTs utilize satellite or aerial sensors to detect water quality parameters by analyzing reflected light.	Large-scale monitoring, non-intrusive.	Resolution limits, data interpretation.
